# Acute Heat Stress Disrupts Hepatic Homeostasis and Gut Microbiota in Early-Juvenile Asian Swamp Eels (*Monopterus albus*)

**DOI:** 10.3390/ani16203206

**Published:** 2026-10-12

**Authors:** Tilin Yi, Kangquan Yu, Jinnan Li, Heng Zhang, Daiqin Yang, Feng Chen

**Affiliations:** 1 Hubei Key Laboratory of Waterlogging Disaster and Agricultural Use of Wetland, College of Animal Science and Technology, Yangtze University, Jingzhou 434000, China; yitilin@163.com (T.Y.);; 2 Jingzhou Taihu Port Aquatic Science and Technology Co., Ltd., Jingzhou 434026, China; 3 Honghu Laboratory, College of Animal Science and Technology, Yangtze University, Jingzhou 434023, China

**Keywords:** *Monopterus albus*, heat stress, liver, gut microbiota, transcriptome

## Abstract

Water temperature is a critical environmental determinant of aquatic organism survival. The Asian swamp eel (*Monopterus albus*), a major aquaculture species, is highly vulnerable to acute summer heat stress at the early-juvenile stage. Juveniles exposed to 32 °C for 96 h (versus 25 °C controls) exhibited 3498 differentially expressed hepatic genes, with enrichment in amino acid metabolism, immune regulation, and apoptosis pathways; immunity, metabolism, and ion binding were top enriched functions. Key genes linked to immune activation, apoptosis, and metabolic reprogramming were upregulated. Gut 16S rRNA profiling revealed increased microbial diversity and taxonomic shifts indicative of microbiota disruption. These findings show that acute heat stress disrupts hepatic homeostasis via coordinated inflammatory, apoptotic, and metabolic responses while altering gut microbiota, offering an integrated view of thermal resilience mechanisms in early-life-stage teleosts.

## Simple Summary

Water temperature is a pivotal environmental driver of survival and physiological homeostasis in aquatic organisms. Integrating hepatic transcriptomic profiling and 16S rRNA gene sequencing of the gut microbiota, we systematically characterized the multi-level impacts of acute heat stress on early-juvenile Asian swamp eels (*Monopterus albus*). Our findings demonstrate that acute heat stress induces hepatic metabolic dysregulation, activation of intrinsic apoptotic pathways, and robust pro-inflammatory immune responses—concurrently triggering significant compositional shifts and increased diversity in the gut microbial community. This study provides the first integrated omics evidence linking thermal stress to coordinated host–microbiota disruption during early juvenile development in the Asian swamp eel, thereby bridging a critical knowledge gap in aquaculture thermal physiology and advancing mechanistic insights into teleost stress resilience.

## 1. Introduction

Water temperature is a pivotal environmental determinant of survival, development, and physiological homeostasis in aquatic organisms [1]. Over recent decades, climate-driven thermal anomalies—including increased frequency and intensity of marine heatwaves—have emerged as critical threats to aquatic ecosystems and global aquaculture productivity [2,3]. As ectothermic vertebrates, fish lack endogenous thermoregulation; consequently, their core body temperature equilibrates directly with ambient water temperature, rendering all biochemical, physiological, and behavioral processes highly temperature-dependent [4]. Acute or chronic heat stress disrupts cellular energy metabolism, impairs immune competence, and induces oxidative damage—cumulatively compromising growth performance, feed conversion efficiency, reproductive output, and disease resistance [5,6,7,8]. These systemic perturbations can culminate in developmental arrest, population decline, or mass mortality under sustained thermal extremes.

The Asian swamp eel (*Monopterus albus*), a teleost fish within the order Synbranchiformes and family Synbranchidae, is a commercially vital aquaculture species across Asia, particularly in China, where annual production reached 355,203 metric tons in 2023 [9]. Its optimal thermal niche for growth and survival spans 25–30 °C [10]. Above this range, individuals exhibit thermoregulatory avoidance behaviors—including burrowing into substrates or seeking shaded microhabitats—to mitigate heat exposure. Sustained temperatures ≥ 32 °C impair feeding motivation and reduce feed intake in cultured populations. Notably, conflicting evidence exists regarding thermal tolerance: Mao et al. [11] reported enhanced growth performance in breeding strains following an 8-day acute heat challenge at 34 °C, whereas Li et al. [12] observed significant declines in both growth rate and survival across breeding and wild strains after chronic exposure to the same temperature for 8 weeks. Critically, despite the larval and juvenile stages exhibiting pronounced thermal sensitivity—attributable to underdeveloped osmoregulatory, immune, and metabolic systems—no integrated physiological or molecular characterization of heat stress responses has been conducted specifically in early-life-stage *M. albus*.

High-throughput sequencing—commonly termed next-generation sequencing (NGS)—has become a cornerstone technology for investigating molecular mechanisms underlying fish growth, immune function, evolutionary adaptation, and environmental resilience [13,14,15]. Nevertheless, hepatic transcriptomic responses to acute heat stress remain poorly characterized in teleosts, particularly in economically relevant species. The liver serves as the central metabolic hub in fish, orchestrating critical physiological processes including nutrient metabolism, biosynthesis of essential biomolecules, and xenobiotic detoxification [16]. Given its pivotal regulatory role, we conducted hepatic transcriptome profiling to systematically dissect the molecular perturbations induced by heat stress in the Asian swamp eel. Concurrently, the gut microbiota constitutes a key modulator of host health, influencing energy harvest, lipid storage, gut barrier integrity, and mucosal immunity [17,18,19]. Accumulating evidence demonstrates that thermal stress acts as a potent environmental disruptor of microbial community structure and function in aquatic organisms [20]. Thus, a comprehensive assessment of heat stress impacts on early-juvenile *M. albus* necessitates integrated analysis of both host transcriptional reprogramming and concomitant gut microbiota remodeling.

In this study, liver tissue was collected from early-juvenile Asian swamp eels following 96 h of acute heat stress (32 °C) and subjected to deep RNA sequencing for transcriptome profiling. Bioinformatic analysis was applied to systematically characterize heat-induced perturbations in hepatic metabolic pathways and immune-related gene networks. Concurrently, gut content samples were processed for 16S rRNA gene amplicon sequencing to assess heat stress-driven shifts in microbial community composition, diversity, and taxonomic structure. Collectively, this integrated approach aims to elucidate the molecular and microbial determinants underlying thermal adaptability in early-life-stage *M. albus*, thereby informing evidence-based strategies for climate-resilient aquaculture management.

## 2. Materials and Methods

### 2.1. Animals

Our experimental animals were obtained from Asian swamp eel juveniles bred at the Asian Swamp Eel Research Institute of Yangtze University. The average body weight was 4.05 ± 0.26 g, and the average body length was 10.20 ± 0.17 cm. The fish were reared in an aquarium system at the Research Center for Aquatic Economic Animals of Yangtze University (Jingzhou, China). The Asian swamp eels were acclimated in the laboratory for 7 days before the experiment began. During the acclimatization period, the Asian swamp eels were provided with commercial pellet feed equivalent to 3% of their body weight daily (Tianbang Feed, Huangshi, Hubei, China). The water temperature was maintained at 25.0 ± 0.2 °C; pH levels ranged from 7.5 to 8.0; and dissolved oxygen levels were kept at 6.5 ± 0.5 mg/L with two-thirds of the water changed daily.

### 2.2. Heat Stress Exposure and Sampling

After acclimation, 90 healthy Asian swamp eels were randomly divided into the control group (CG) and acute heat stress group (HG). Each group had three replicate tanks with 15 individuals per tank, and each tank contained 100 L of aerated freshwater. The water temperature was continuously maintained using a thermostatic heating system. The control group was maintained at 25 °C throughout the trial. In the acute heat stress group, the Asian swamp eels were placed in water at 25 °C, and the water temperature was increased from 25 °C to 32 °C at a rate of 1 °C/h using a water temperature control system, then held steadily at 32 °C. The water in all tanks was constantly aerated using air stones, and no food was provided during the test period. Except for water temperature, all other conditions were identical between groups (pH 7.5–8.0, dissolved oxygen 6.5 ± 0.5 mg/L). After exposure to heat stress for 96 h, all fish survived. Three fish were randomly selected from each water tank, anesthetized with MS-222 (100 mg/L), and dissected; their liver and gut contents were collected, pooled by tank, rapidly frozen in liquid nitrogen, and stored at −80 °C until hepatic transcriptome analysis and gut microbiota analysis were conducted.

### 2.3. Transcriptome Analysis

#### 2.3.1. RNA Extraction, Library Construction, and Sequencing

Total RNA was isolated from the liver samples using TRIzol Reagent (Invitrogen Life Technologies, Carlsbad, CA, USA), and the concentration, quality, and integrity were determined using a NanoDrop spectrophotometer (Thermo Scientific, Carlsbad, CA, USA). The mRNA was purified from total RNA using poly-T oligo-attached magnetic beads. First-strand cDNA was synthesized using random oligonucleotides and SuperScript II. Subsequently, second-strand cDNA synthesis was performed using DNA Polymerase I and RNase H. To select cDNA fragments of the preferred length of 400–500 bp, the library fragments were purified using the AMPure XP system (Beckman Coulter, Beverly, CA, USA). DNA fragments with ligated adaptor molecules on both ends were selectively enriched using Illumina PCR Primer Cocktail in a 15-cycle PCR. The products were then purified (AMPure XP system) and quantified using the Agilent high-sensitivity DNA assay on a Bioanalyzer 2100 system (Agilent, Santa Clara, CA, USA). After RNA extraction and library construction, sequencing was performed on the NovaSeq 6000 platform (Illumina, San Diego, CA, USA) by Bioyi Biotechnology Co., Ltd., Wuhan, China. Raw RNA-seq data have been submitted to the Sequence Read Archive (SRA) database at NCBI (accession number: SRP352198).

#### 2.3.2. Bioinformatics Analyses

Clean reads were obtained by using Cutadapt (v1.15) to remove sequences with adapters at the 3′ end from the raw reads and also removing reads with an average quality score below Q20 (representing a sequencing error rate of ≤1%). Indices of the reference genome (GCF_001952655.1) were constructed using HISAT2 (v2.0.5), and paired-end clean reads were aligned to the reference genome using HISAT2 (v2.0.5) and further analyzed and annotated based on the reference genome. To ensure comparability of gene expression levels across different genes and samples, we normalized the expression levels using TPM (transcripts per million). Gene expression was analyzed using DESeq (v1.39.0), with screening criteria for differentially expressed genes set as follows: |log_2_FoldChange| > 1 for fold difference in expression and an adjusted *p* value < 0.05 after FDR correction. The number of differentially expressed genes after screening was counted, and volcano plots of these genes were drawn using the R package ggplot2 (v4.0.3). The gene ontology (GO) enrichment analysis was performed using topGO, with *p* value calculated by the hypergeometric distribution method (significant enrichment criterion: adjusted *p* value < 0.05). Significantly enriched GO terms identified through this analysis helped determine the main biological functions carried out by the differentially expressed genes. KEGG pathway enrichment analysis focused on significantly enriched pathways with an adjusted *p* value < 0.05, utilizing the ClusterProfiler (v3.4.4) software package for this purpose.

### 2.4. Gut Microbiota Analysis

#### 2.4.1. DNA Extraction, PCR Amplification, and Sequencing

Gut microbial DNA was extracted from the gut contents of Asian swamp eels using the Omega Soil DNA Kit (D5625-01) (Omega Bio-Tek, Norcross, GA, USA). The concentration of gDNA samples was evaluated using a NanoDrop spectrophotometer (Thermo Fisher Scientific, Carlsbad, CA, USA). The V3–V4 region of the 16S rDNA gene was amplified using primers 338F (5′-barcode + ACTCCTACGGGAGGCAGCA-3′) and 806R (5′-GGACTACHVGGGTWTCTAAT-3′), with barcode sequences incorporated into the forward primer. PCR products were purified. Sequencing libraries were prepared using the TruSeq Nano DNA LT Library Prep Kit from Illumina. For eligible libraries, we performed 2 × 250 bp paired-end sequencing on an Illumina NovaSeq instrument using the NovaSeq 6000 SP Reagent Kit (500 cycles). Raw 16S rRNA gene sequencing data have been submitted to the SRA database at NCBI (accession number: SRP362364).

#### 2.4.2. Biodiversity Analysis

Microbiome bioinformatics were performed using QIIME2 2022.11 [21] according to the official tutorials (https://docs.qiime2.org/2022.11/tutorials/ (accessed on 10 May 2024)). The raw data were preprocessed, quality filtered, denoised, trimmed, and dechimerized to minimize noise following the DADA2 (v1.26) method using the default parameters [22]. The above sequences were clustered at 100% sequence identity to generate amplicon sequence variants (ASVs). ASVs were aligned against reference sequences from the Greengenes database (http://greengenes.secondgenome.com/ (accessed on 10 May 2024)) to obtain taxonomic information for each ASV. Using QIIME2 software, we calculated seven alpha diversity indicators after setting the rarefaction depth to 95% of the minimum sample sequence count, including Chao1, Simpson, Shannon, Pielou’s evenness, Observed species, Faith’s phylogenetic diversity (PD), and Good’s coverage index, and visualized them as boxplots. Beta diversity assessment included principal coordinates analysis (PCoA) and the unweighted pair-group method with arithmetic means (UPGMA). The significance of differentiation of microbiota structure among groups was assessed by PERMANOVA (Permutational multivariate analysis of variance) using QIIME2. Venn diagrams were generated in R to enumerate unique and shared ASVs across groups, regardless of their relative abundance. All data were assessed for normality and homogeneity of variance prior to further analysis; where necessary, data transformations were applied to meet the assumptions of analysis of variance. Differences were considered significant at *p* < 0.05.

## 3. Results

### 3.1. Transcriptome Sequencing

Six cDNA libraries were constructed and sequenced from livers of the Asian swamp eel subjected to distinct temperature treatments for 96 h. Library characteristics are summarized in Table 1. A total of 297,035,056 raw reads were generated across the six experimental groups. Following quality filtering, 277,926,908 high-quality clean reads remained—representing an average retention rate of 93.57% of raw reads. All libraries achieved Q30 scores exceeding 93.94%, indicating robust base-calling accuracy (i.e., ≥99.9% probability per base). These clean reads were used for downstream transcriptomic analysis; on average, 84.69% (±2.12%) aligned uniquely to the *Monopterus albus* reference genome, while an additional 6.57% (±0.23%) mapped to multiple genomic loci.

### 3.2. Identification and Enrichment Analyses of Differentially Expressed Genes

**Figure 1:**
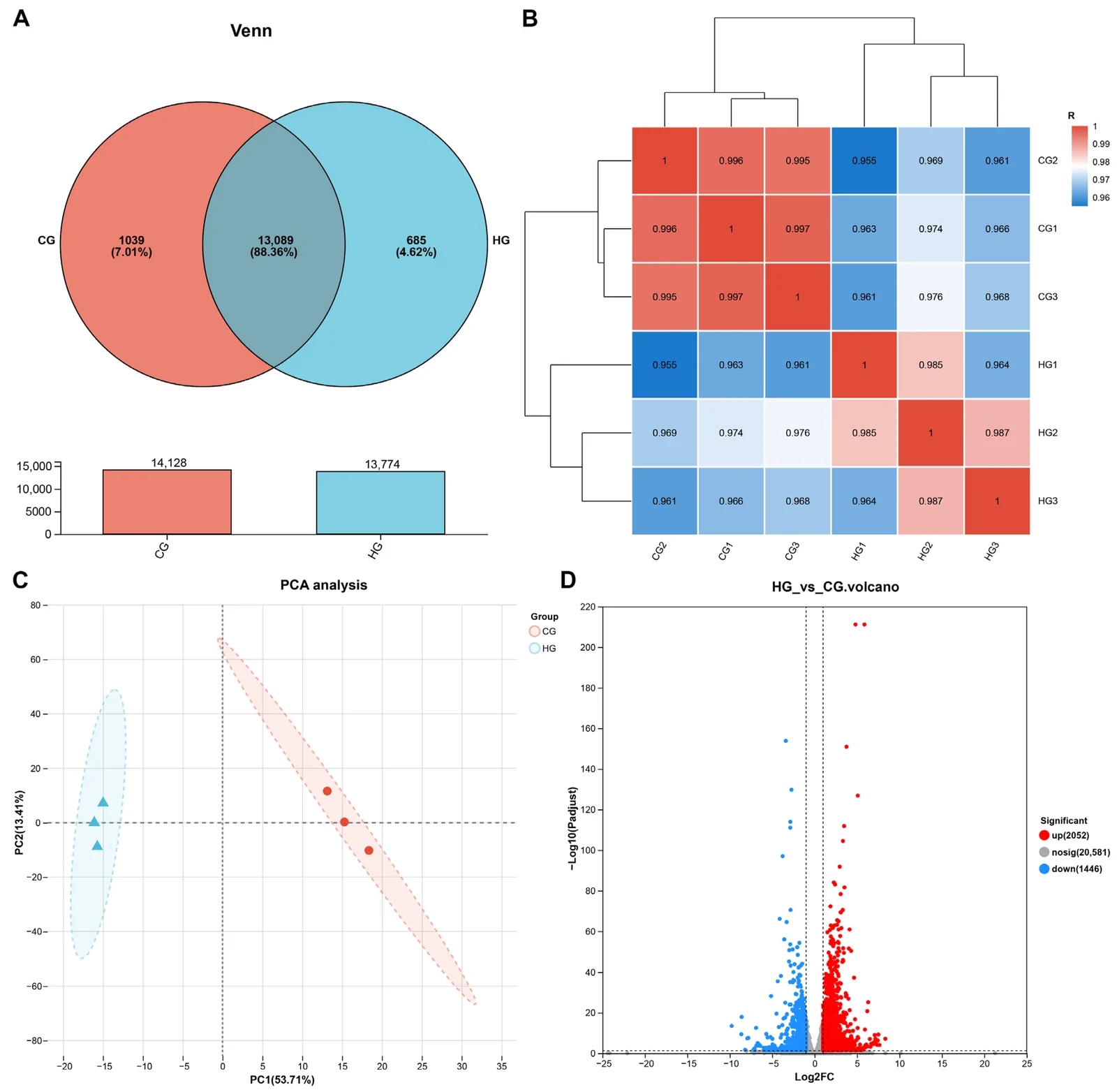
Analyses of sample relationships and expression level differences. (**A**) Circles of different colors represent genes selected based on expression levels within a group, with the numbers indicating the count of shared and unique genes among different groups. (**B**) Different colored squares represent the degree of correlation between two samples. (**C**) After dimensionality reduction analysis, the samples have relative coordinate points on the principal components, where the distance between each pair of sample points represents their dissimilarity; the closer the distance, the higher the similarity between the samples. (**D**) The *x*-axis represents the fold change in gene expression between the two samples. The *y*-axis shows the statistical significance of the gene expression difference, represented by the adjusted *p* value. Red dots indicate significantly upregulated genes, blue dots represent significantly downregulated genes, and gray dots denote non-significantly differentially expressed genes.

Gene expression profiling revealed that the control group (CG) and heat-stressed group (HG) expressed 14,128 and 13,774 genes, respectively (Figure 1A). These groups shared 13,089 commonly expressed genes, with 1039 genes uniquely expressed in CG and 685 in HG. Both Pearson correlation analysis and principal component analysis (PCA) confirmed that inter-group variation exceeded intra-group variation (Figure 1B,C). Differential expression analysis using DESeq identified 3498 differentially expressed genes (DEGs) in liver tissue when comparing the heat-stressed group with the control group, comprising 2052 upregulated and 1446 downregulated genes (Figure 1D). Applying an adjusted *p* value threshold of <0.05, Table 2 lists the top 22 most significantly altered DEGs (|log_2_FoldChange| > 4).

**Figure 2:**
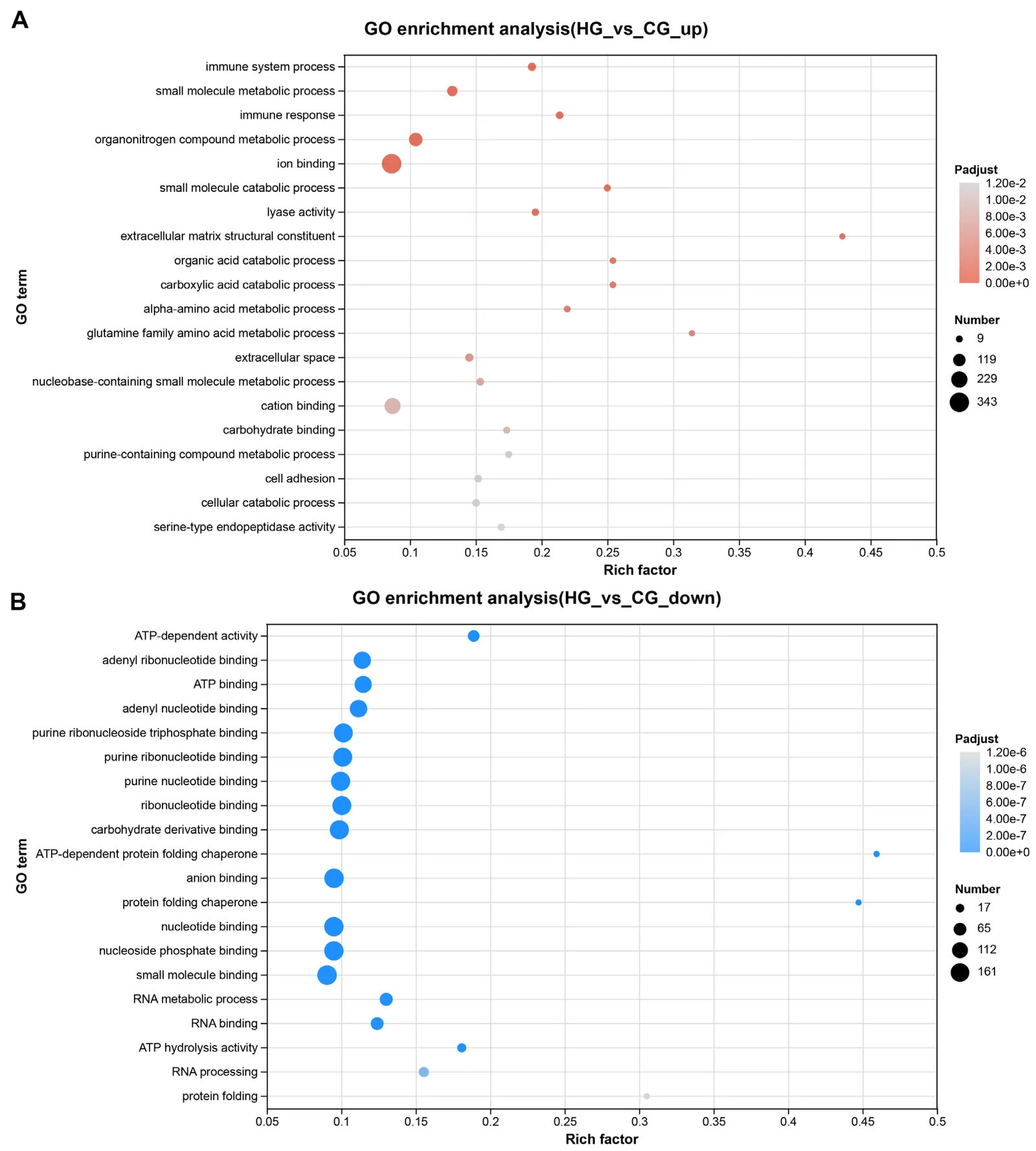
GO enrichment analysis of upregulated (**A**) and downregulated (**B**) genes. The *y*-axis represents GO terms; the *x*-axis represents the Rich factor. The size of each point corresponds to the number of genes within that GO term, and the color of the points reflects different ranges of adjusted *p* values.

GO enrichment analysis was performed separately for upregulated and downregulated genes. Results revealed that upregulated genes were significantly enriched in biological processes related to immunity (e.g., immune system process and immune response), metabolism (e.g., small molecule metabolic process, organonitrogen compound metabolic process, and small molecule catabolic process), and molecular functions involving ion binding (Figure 2A). In contrast, downregulated genes were predominantly enriched in ATP-related molecular functions (e.g., ATP-dependent activity, ATP binding, and ATP hydrolysis activity), nucleotide-binding activities (e.g., adenyl ribonucleotide binding, adenyl nucleotide binding, and purine ribonucleoside triphosphate binding), and protein folding-associated functions (e.g., ATP-dependent protein folding chaperone, protein folding chaperone, and protein folding) (Figure 2B).

**Figure 3:**
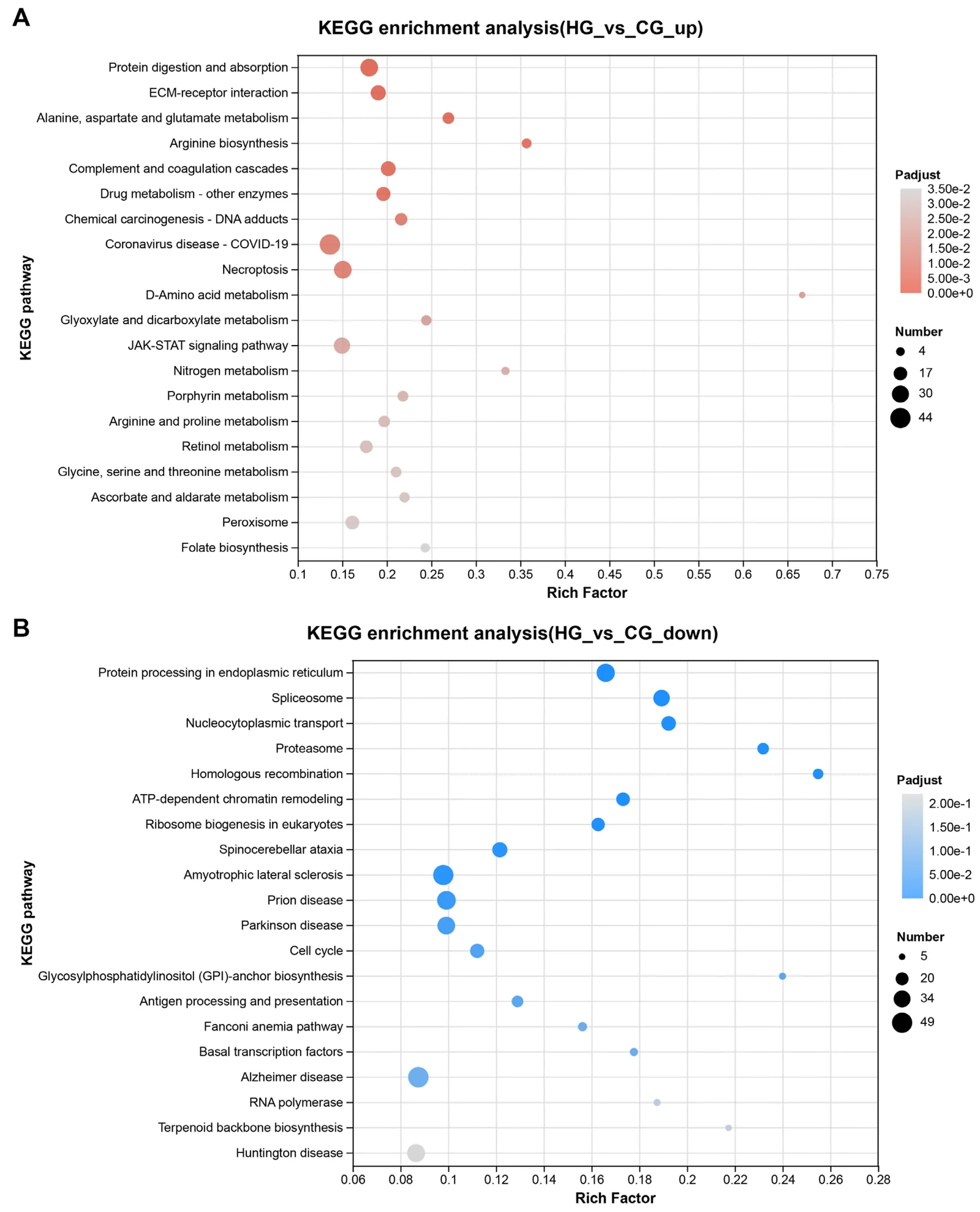
KEGG pathway analysis of upregulated (**A**) and downregulated (**B**) genes. The *y*-axis represents pathway names; the *x*-axis represents the Rich factor. The size of the points reflects the number of genes in each pathway, and the color of the points corresponds to different adjusted *p* value ranges.

KEGG pathway enrichment analysis was conducted separately for upregulated and downregulated genes. Results demonstrated that upregulated genes were significantly enriched in pathways related to amino acid metabolism (e.g., protein digestion and absorption; alanine, aspartate and glutamate metabolism; arginine biosynthesis), immune regulation (e.g., ECM-receptor interaction; complement and coagulation cascades), and programmed cell death (e.g., necroptosis) (Figure 3A). In contrast, downregulated genes were predominantly enriched in pathways associated with protein synthesis and homeostasis (e.g., protein processing in the endoplasmic reticulum; nucleocytoplasmic transport; proteasome; ribosome biogenesis in eukaryotes) and DNA metabolism and integrity maintenance (e.g., spliceosome; homologous recombination; ATP-dependent chromatin remodeling) (Figure 3B).

### 3.3. Microbial Composition and Diversity Analyses

**Figure 4:**
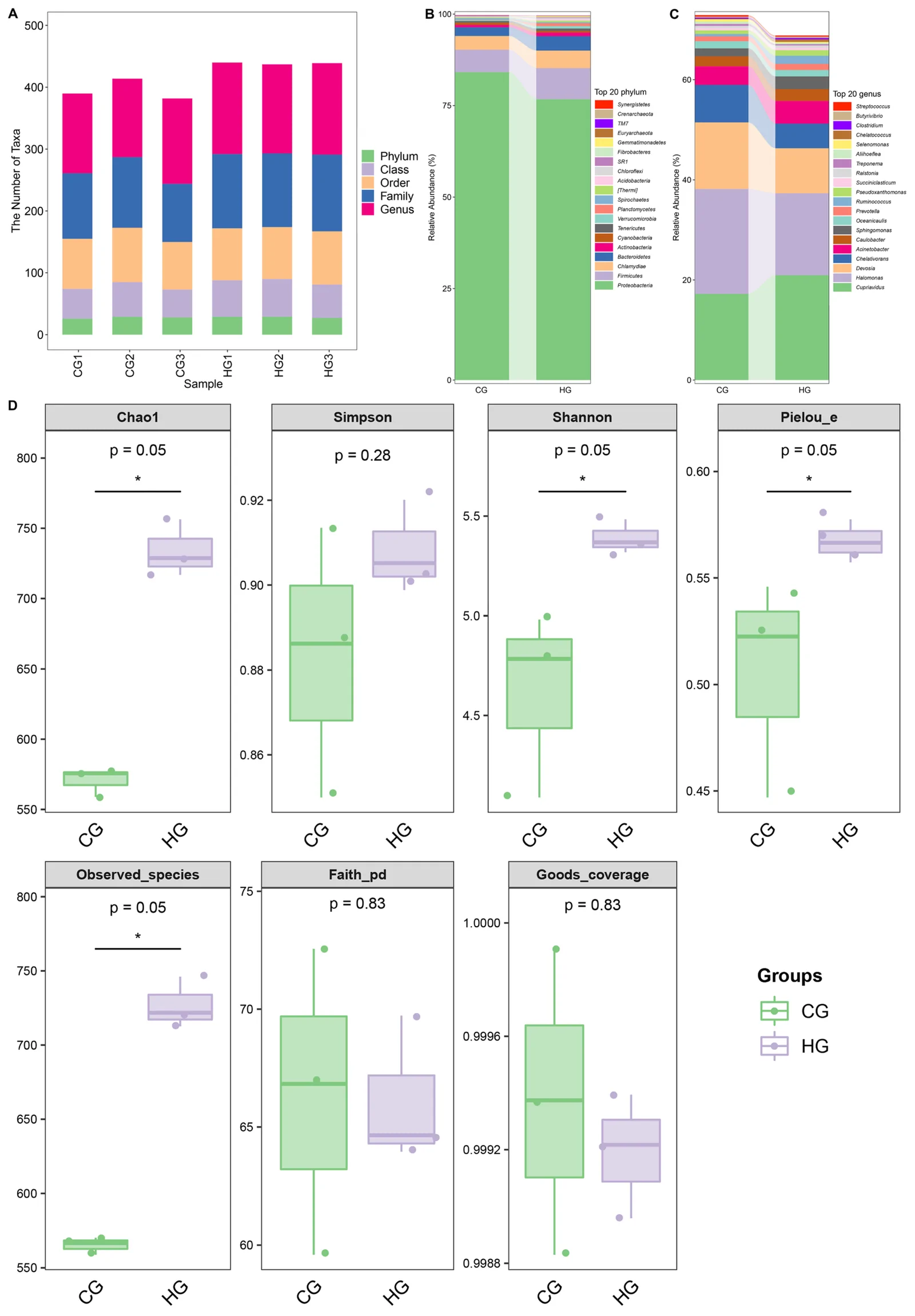
Gut microbial composition and diversity analyses. (**A**) Taxonomic profiling across classification levels for each sample. (**B**) Relative abundance at the phylum level. (**C**) Relative abundance at the genus level. (**D**) Boxplot of alpha diversity indices by group. The numbers under the diversity index label are the *p* values from the Kruskal–Wallis test. An asterisk indicates a significant difference.

A total number of 298,136 high-quality sequences were recovered from all gut samples (Table 3), with a mean read length of 207 bp. Taxonomic profiling across classification levels revealed that the HG group harbored significantly greater richness—i.e., higher total numbers of taxonomic units—than the CG group (Figure 4A). At the phylum level, the four most abundant taxa were Proteobacteria, Firmicutes, Chlamydiae, and Bacteroidetes, in descending order of relative abundance (Figure 4B). At the genus level, the top five most abundant taxa were *Cupriavidus*, *Halomonas*, *Devosia*, *Chelativorans*, and *Acinetobacter*, respectively (Figure 4C). Alpha diversity analysis demonstrated that the HG group exhibited significantly higher values for the Chao1 index, Shannon index, Pielou’s evenness index, and observed species index compared with the CG group (Figure 4D). In contrast, no significant differences were observed for the Simpson index, Faith’s phylogenetic diversity (PD) index, or Good’s coverage index.

### 3.4. Microbial Species Difference Analysis

**Figure 5:**
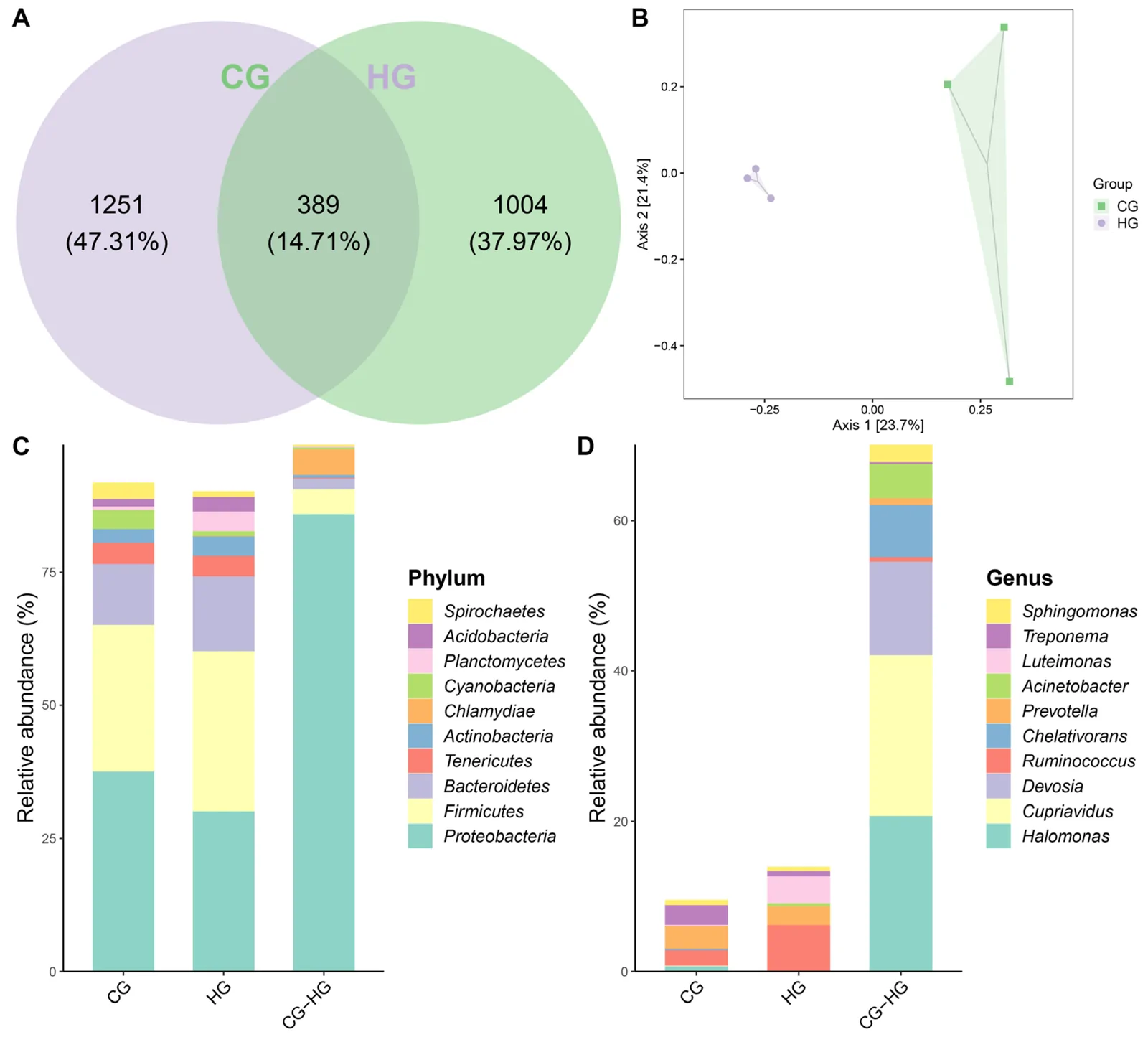
Gut microbial species difference analysis. (**A**) Venn plot of ASV counts. (**B**) Two-dimensional ordination plot of PCoA analysis of beta diversity for samples. (**C**,**D**) Bar chart of ASV abundance across different regions of the Venn diagram.

A total of 3033 ASVs (not their abundance values) were recovered across all samples, of which 389 ASVs were shared between the CG and HG groups (Figure 5A). The CG and HG groups harbored 1251 and 1004 unique ASVs, respectively. Principal coordinates analysis (PCoA) based on weighted UniFrac distance revealed clear separation between the two groups: the three biological replicates from the HG group clustered tightly together, while remaining distinctly separated from the three replicates of the CG group (Figure 5B). The result of the PERMANOVA statistical test indicates a significant difference in gut microbial community structure between the HG and CG groups.

Relative abundances of dominant microbial taxa at the phylum and genus levels are presented in Figure 5C,D. At the phylum level, Proteobacteria, Firmicutes, Bacteroidetes, Tenericutes, and Actinobacteria were the five most abundant phyla across all gut samples (Figure 5C). Subdominant phyla included Chlamydiae, Cyanobacteria, Planctomycetes, Acidobacteria, and Spirochaetes. Comparative analysis revealed significantly higher relative abundances of Bacteroidetes, Actinobacteria, Planctomycetes, and Acidobacteria in the HG group than in the CG group; conversely, Proteobacteria, Cyanobacteria, and Spirochaetes were significantly more abundant in the CG group. Chlamydiae exhibited consistently high relative abundance in both groups. At the genus level, *Ruminococcus* and *Prevotella* were the most prevalent genera in the Asian swamp eel gut microbiota, followed by *Treponema*, *Luteimonas*, and *Sphingomonas* (Figure 5D). *Ruminococcus*, *Luteimonas*, and *Acinetobacter* showed significantly higher relative abundances in the HG group, whereas *Treponema*, *Halomonas*, and *Prevotella* were significantly enriched in the CG group. *Halomonas*, *Cupriavidus*, *Devosia*, *Chelativorans*, and *Acinetobacter* were highly abundant in both groups.

## 4. Discussion

Thermal stress constitutes a primary abiotic stressor in fish physiology research. Under acute heat stress, teleosts mount a conserved physiological response characterized by elevated oxygen consumption and reallocation of energetic resources—diverting ATP from anabolic processes such as growth and reproduction toward cellular maintenance and stress defense. This metabolic prioritization inevitably compromises immune competence and increases susceptibility to opportunistic pathogens [23]. Although the liver serves as the central metabolic and detoxification organ in fish, molecular responses to acute thermal challenge remain undercharacterized at the transcriptomic level, particularly in non-model aquaculture species [24,25]. In this study, we applied integrated hepatic transcriptomics and gut 16S rRNA gene profiling to dissect the host–microbiota axis perturbed by environmentally relevant acute heat stress in early-juvenile Asian swamp eel, thereby elucidating coordinated molecular and microbial mechanisms underlying thermal response.

Hepatic transcriptome analysis of early-juvenile Asian swamp eel exposed to acute heat stress revealed that upregulated DEGs were significantly enriched in metabolic biological processes—including small molecule metabolic process, organonitrogen compound metabolic process, and small molecule catabolic process—as well as in KEGG pathways such as protein digestion and absorption; alanine, aspartate and glutamate metabolism; and arginine biosynthesis. This enrichment pattern indicates that thermal challenge induces a systemic reconfiguration of hepatic metabolic networks rather than isolated pathway alterations. Among the top DEGs, cysteine three histidine 1 (*cth1*), glutamate receptor metabotropic 6b (*grm6b*), elongation of very long chain fatty acids protein 6 (*elovl6*), ceramide kinase (*cerk*), and glutamate receptor metabotropic 8b (*grm8b*) emerged as central metabolic regulators. Consistent with this, *cth1*, *grm6b*, and *elovl6* were significantly downregulated, whereas *cerk* and *grm8b* were significantly upregulated. Integrated functional interpretation reveals a potential coherent adaptive strategy: suppression of *cth1* possibly promotes metabolic flexibility and stress resilience [26]; reduced *grm6b* expression likely alleviates inhibition of cAMP/PKA signaling, thereby facilitating rapid energy mobilization [27]; *elovl6* downregulation may shift hepatic lipid composition toward increased saturation, contributing to enhanced glucose production and insulin resistance [28]; *cerk* upregulation possibly drives ceramide accumulation—a well-established mediator of lipotoxicity and mitochondrial dysfunction in metabolic stress [29,30]; and *grm8b* induction may modulate hypothalamic–pituitary–interrenal (HPI) axis activity, reflecting conserved neuroendocrine engagement during thermal challenge in teleosts [31]. Together, these transcriptional changes orchestrate an integrated increase in hepatic metabolic capacity to meet the escalated energetic demands of cellular thermoregulation and proteostasis maintenance, which is similar to catfish [32] and smelt fish [33].

Acute heat stress—imposed as an external environmental challenge—can disrupt physiological homeostasis and elicit a pro-inflammatory immune shift in ectothermic vertebrates. This study revealed that upregulated DEGs were significantly enriched in immune-related biological processes (e.g., immune system process and immune response) and KEGG pathways (e.g., ECM-receptor interaction; complement and coagulation cascades). Among the top-ranked DEGs, canonical immune effectors—including Toll-like receptor 7 (*tlr7*), DNA nucleotidylexotransferase (*dntt*), and regucalcin (*rgn*)—were robustly induced. These findings collectively indicate a transcriptionally coordinated activation of both innate and adaptive immune components, consistent with immune priming responses documented in teleosts under thermal stress [34,35,36]. Specifically, *tlr7* encodes a key pattern-recognition receptor that initiates antiviral type I interferon responses upon sensing viral ssRNA; its upregulation suggests potentially heightened surveillance against pathogen-associated molecular patterns during thermal challenge [37]. DNA nucleotidylexotransferase (*DNTT*) is indispensable for V(D)J recombination at the T-cell receptor (TCR) locus, thereby enabling T-cell receptor diversity and clonal expansion—a prerequisite for adaptive immunity [38]. T cells not only mediate host defense against intracellular pathogens but also modulate inflammatory resolution, metabolic homeostasis, and tumor immunosurveillance. Regucalcin (*rgn*), first identified in 1978 as a non-EF-hand Ca^2+^-binding protein, functions as a multifunctional regulator of intracellular Ca^2+^ homeostasis, antioxidant defense, and apoptosis suppression—processes critically linked to immune cell survival and function [39,40]. The concurrent upregulation of *tlr7*, *dntt*, and *rgn* may imply a concerted enhancement of pathogen recognition, lymphocyte diversification, and immune cell resilience under acute heat stress. Notably, H1 histone family member 0 (*h1f0*), recently implicated as a stress-responsive epigenetic modulator associated with chronic inflammation and metabolic dysregulation in vertebrates [41], was among the most strongly induced transcripts. Its marked upregulation reflects transcriptional reprogramming beyond acute immune activation—potentially signifying early chromatin-level adaptations that may influence long-term immunometabolic outcomes, warranting further mechanistic investigation.

Apoptosis—a highly regulated form of programmed cell death—is activated in response to diverse physiological cues and pathological insults, including thermal stress [42,43]. Accumulating evidence demonstrates that elevated temperature disrupts cellular proteostasis and mitochondrial integrity, thereby triggering intrinsic apoptotic pathways in teleost hepatocytes [44]. Consistent with this, heat stress has been shown to induce apoptosis in the liver across multiple fish species, including largemouth bass (*Micropterus salmoides*) [45], pikeperch (*Sander lucioperca*) [7], rainbow trout (*Oncorhynchus mykiss*) [46,47], *Brachymystax lenok tsinlingensis* [48], spotted seabass (*Lateolabrax maculatus*) [49], giant clam (*Tridacna crocea*) [50], and grass carp (*Ctenopharyngodon idellus*) [51]. In the present study, acute heat stress significantly altered the hepatic expression of key apoptosis-regulatory genes in Asian swamp eel, notably dual specificity phosphatase 13a (*dusp13a*) and ADAM metallopeptidase with thrombospondin type 1 motif 14 (*adamts14*). DUSP13A physically interacts with the N-terminal regulatory domain of ASK1 (apoptosis signal-regulating kinase 1), relieving autoinhibitory constraints and thereby promoting ASK1-dependent activation of the JNK/p38–caspase-3 cascade [52]. Similarly, *adamts14* overexpression has been experimentally linked to caspase-dependent apoptosis induction, suppression of cell proliferation, and inhibition of extracellular matrix remodeling—processes collectively indicative of pro-apoptotic and anti-proliferative activity in epithelial and mesenchymal contexts [53]. Together, these transcriptional changes provide convergent molecular evidence for the activation of intrinsic apoptotic signaling in the liver of Asian swamp eel following acute heat exposure.

Furthermore, acute heat stress induced significant compositional shifts in the gut microbiota of Asian swamp eel in both microbial composition and species difference analyses. At the phylum level, Proteobacteria, Firmicutes, and Bacteroidetes constituted the dominant bacterial taxa—consistent with prior profiling of this species’ gut microbiota, which identified Firmicutes, Fusobacteria, Proteobacteria, Bacteroidetes, and Actinobacteria as the five most abundant phyla [54]. Notably, heat exposure significantly reduced the relative abundance of Proteobacteria and Cyanobacteria and markedly decreased the Firmicutes-to-Bacteroidetes (F/B) ratio. Given that the F/B ratio is a well-documented microbial signature associated with host energy harvest, lipid metabolism, and adiposity across vertebrates [55,56], its reduction under acute thermal stress suggests a potential perturbation of gut energy homeostasis—possibly contributing to transient catabolic states or reduced nutrient retention efficiency in the host. At the class and order levels, Planctomycetia—including members of the order Planctomycetales—were significantly enriched in heat-stressed individuals. In Atlantic salmon (*Salmo salar*), Planctomycetes enhance dietary carbohydrate digestion via extracellular glycoside hydrolases [57]; similarly, in common carp (*Cyprinus carpio*), they contribute to polysaccharide degradation and participate in host-associated nitrogen cycling through amino acid fermentation and ammonium assimilation [58,59]. The observed enrichment of Planctomycetia possibly implies a heat-induced functional adaptation toward enhanced carbohydrate catabolism—potentially supporting rapid energy mobilization during thermal challenge. At the genus level, *Ruminococcus* and *Prevotella* emerged as dominant taxa. *Ruminococcus* spp. are proficient degraders of resistant starch and other complex plant-derived polysaccharides in anaerobic gut environments [60], whereas *Prevotella* spp. modulate mucosal immunity and have been shown to attenuate experimental colitis and arthritis in murine models via short-chain fatty acid-dependent and Treg-mediated mechanisms [61]. Strikingly, *Luteimonas*—a genus capable of fermenting amino acids to produce acetate and propionate [62]—was significantly upregulated under heat stress. This shift suggests potentially increased reliance on protein-derived carbon substrates for microbial energy generation, possibly reflecting host proteolysis or dietary protein reallocation under energetic demand. Collectively, these taxonomic and inferred functional alterations indicate that acute heat stress restructures the gut microbiota of Asian swamp eel in ways that likely influence host metabolic regulation—particularly energy substrate utilization, immune-microbe crosstalk, and nitrogen metabolism—thereby representing a previously underappreciated axis of thermal acclimation.

Although integrating liver transcriptome and gut microbiome data revealed that acute heat stress disrupts hepatic homeostasis and gut microbiota in early-juvenile Asian swamp eel, this study still has limitations. First, physiological indicators associated with heat stress—such as cortisol levels, oxygen consumption, and blood parameters—are missing. These indicators could provide a more comprehensive understanding of how fish respond to heat stress. Second, there is a lack of experimental validation for key genes and microbes involved in fish responses to heat stress. Such experiments would offer crucial insights for stress-resistant breeding and the development of microbial products. Finally, since the main production regions for Asian swamp eel in China are along the Yangtze River basin, where high temperatures persist for extended periods, chronic or subchronic heat stress experiments would be more relevant for guiding artificial aquaculture practices.

## 5. Conclusions

In conclusion, integrated hepatic transcriptomics and gut 16S rRNA sequencing of early-juvenile Asian swamp eel exposed to acute heat stress revealed three coordinated responses: (1) 3498 DEGs, with KEGG enrichment showing disruption in amino acid metabolism, immune regulation, and apoptosis pathways; GO analysis confirmed top enrichments in immunity, metabolism, and ion binding; (2) consistent upregulation of key DEGs driving immune activation, apoptosis execution, and metabolic reprogramming; and (3) significant gut microbiota shifts—including increased alpha diversity and altered abundances of core taxa—indicating heat-induced microbiota disruption. These multi-omics signatures demonstrate that acute heat stress triggers a systemic, multiorgan response: hepatic metabolic–immune–apoptotic crosstalk and gut microbiota-mediated modulation of nutrient processing and immune priming. This work provides the first mechanistic, multi-layered characterization of thermal stress resilience in early-life-stage *M. albus*, establishing a foundational framework for understanding teleost acclimation to rising aquatic temperatures.

## Figures and Tables

**Table 1:** Summary statistics of the transcriptome sequences.

Sample	Row Reads	Clean Reads	Clean Bases	Q30 (%)	Total Mapped Reads (Ratio)	Multiple Mapped Reads (Ratio)	Uniquely Mapped Reads (Ratio)
CG1	51,556,178	48,092,848	7,733,426,700	94.38%	41,163,659 (85.59%)	2,706,801 (6.58%)	38,456,858 (93.42%)
CG2	45,604,968	42,619,898	6,840,745,200	94.18%	36,490,519 (85.62%)	2,404,395 (6.59%)	34,086,124 (93.41%)
CG3	45,100,746	42,220,644	6,765,111,900	94.48%	36,270,132 (85.91%)	2,382,873 (6.57%)	33,887,259 (93.43%)
HG1	53,522,510	49,999,274	8,028,376,500	93.94%	41,282,098 (82.57%)	2,758,612 (6.68%)	38,523,486 (93.32%)
HG2	52,211,248	48,893,414	7,831,687,200	94.02%	41,082,551 (84.02%)	2,728,405 (6.64%)	38,354,146 (93.36%)
HG3	49,039,406	46,100,830	7,355,910,900	94.36%	38,934,211 (84.45%)	2,467,538 (6.34%)	36,466,673 (93.66%)

Note: Q30 (%) indicates the percentage of bases with a base calling accuracy of more than 99.9%.

**Table 2:** The 22 most significantly differentially expressed genes between the CG and HG groups (|log_2_FoldChange| > 4).

Gene ID	Gene Name	Change	log_2_Fold Change	pval	padj	Description
ENSMALG00000020738	*cth1*	down	−5.4959	0.0102	0.0392	cysteine three histidine 1
ENSMALG00000020706	*heyl*	down	−5.1889	0.0010	0.0054	hes-related family bHLH transcription factor with YRPW motif like
ENSMALG00000007898	*grm6b*	down	−5.1010	9.4882 × 10^−36^	3.2854 × 10^−33^	glutamate receptor, metabotropic 6b
ENSMALG00000013294	*myo1ha*	down	−4.5241	0.0098	0.0379	myosin IHa
ENSMALG00000020855	*dynll1*	down	−4.4540	5.6626 × 10^−6^	6.0685 × 10^−5^	dynein, light chain, LC8-type 1
ENSMALG00000009702	*elovl6*	down	−4.3052	1.5659 × 10^−22^	1.8831 × 10^−20^	ELOVL fatty acid elongase 6
ENSMALG00000014349	*nrf1*	down	−4.0517	8.6311 × 10^−67^	1.5758 × 10^−63^	nuclear respiratory factor 1
ENSMALG00000015154	*rab25a*	down	−4.0425	1.2422 × 10^−6^	1.5641 × 10^−5^	RAB25, member RAS oncogene family a
ENSMALG00000019406	*ddc*	up	4.0475	4.3639 × 10^−36^	1.5375 × 10^−33^	dopa decarboxylase
ENSMALG00000015408	*slc26a10*	up	4.1052	6.0269 × 10^−21^	5.8757 × 10^−19^	solute carrier family 26 member 10
ENSMALG00000004070	*btbd3b*	up	4.2578	0.0002	0.0017	BTB (POZ) domain containing 3b
ENSMALG00000002200	*rgn*	up	4.2915	1.7742 × 10^−92^	1.1877 × 10^−88^	regucalcin
ENSMALG00000000487	*alk*	up	4.3454	2.4201 × 10^−5^	0.0002	ALK receptor tyrosine kinase
ENSMALG00000008012	*cerk*	up	4.6943	3.4137 × 10^−42^	1.7140 × 10^−39^	ceramide kinase
ENSMALG00000009113	*plcd3b*	up	4.7882	4.0606 × 10^−20^	3.6406 × 10^−18^	phospholipase C, delta 3b
ENSMALG00000015174	*dusp13a*	up	4.8491	5.6849 × 10^−8^	9.5699 × 10^−7^	dual specificity phosphatase 13a
ENSMALG00000009843	*DNTT*	up	4.8927	2.9102 × 10^−5^	0.0003	DNA nucleotidylexotransferase
ENSMALG00000001449	*adamts14*	up	5.0108	1.0566 × 10^−5^	0.0001	ADAM metallopeptidase with thrombospondin type 1 motif, 14
ENSMALG00000012420	*tlr7*	up	5.0337	0.0073	0.0301	toll-like receptor 7
ENSMALG00000021085	*nkx2.1*	up	5.4753	0.0113	0.0427	NK2 homeobox 1
ENSMALG00000014374	*grm8b*	up	5.4971	5.2276 × 10^−5^	0.0004	glutamate receptor, metabotropic 8b
ENSMALG00000001717	*h1f0*	up	5.9367	1.6269 × 10^−150^	1.6336 × 10^−146^	H1 histone family, member 0

**Table 3:** Characteristics of full-length 16S rRNA sequences from Asian swamp eel gut microbial samples.

Sample ID	Raw Sequences	Clean Sequences	Effective Sequences	Effective (%)
CG1	63,547	57,610	57,163	89.95
CG2	43,221	39,100	38,704	89.55
CG3	56,291	51,410	51,048	90.69
HG1	60,151	55,005	54,548	90.69
HG2	50,595	46,086	45,611	90.15
HG3	56,143	51,478	51,062	90.95

## Data Availability

The original data presented in the study are openly available in the SRA database in NCBI (accession numbers: SRP352198 and SRP362364). Further inquiries can be directed to the corresponding author.
